# Extreme Terrestrial Environments: Life in Thermal Stress and Hypoxia. A Narrative Review

**DOI:** 10.3389/fphys.2018.00572

**Published:** 2018-05-16

**Authors:** Martin Burtscher, Hannes Gatterer, Johannes Burtscher, Heimo Mairbäurl

**Affiliations:** ^1^Department of Sport Science, University of Innsbruck, Innsbruck, Austria; ^2^Austrian Society for Alpine and Mountain Medicine, Innsbruck, Austria; ^3^Institute of Mountain Emergency Medicine, EURAC Research, Bolzano, Italy; ^4^Laboratory of Molecular and Chemical Biology of Neurodegeneration, École Polytechnique Fédérale de Lausanne, Lausanne, Switzerland; ^5^Medical Clinic VII, Sports Medicine, University Hospital Heidelberg, Heidelberg, Germany; ^6^German Center for Lung Research (DZL/TLRC-H), Heidelberg, Germany

**Keywords:** heat, cold, high altitude, natives, sojourners, pre-acclimatization

## Abstract

Living, working and exercising in extreme terrestrial environments are challenging tasks even for healthy humans of the modern new age. The issue is not just survival in remote environments but rather the achievement of optimal performance in everyday life, occupation, and sports. Various adaptive biological processes can take place to cope with the specific stressors of extreme terrestrial environments like cold, heat, and hypoxia (high altitude). This review provides an overview of the physiological and morphological aspects of adaptive responses in these environmental stressors at the level of organs, tissues, and cells. Furthermore, adjustments existing in native people living in such extreme conditions on the earth as well as acute adaptive responses in newcomers are discussed. These insights into general adaptability of humans are complemented by outcomes of specific acclimatization/acclimation studies adding important information how to cope appropriately with extreme environmental temperatures and hypoxia.

## General Aspects of Adapting to Extreme Environments

Coping with extreme environments is challenging. It is not just survival in extreme terrestrial environments, but rather the necessity for performance in everyday life, occupation and sports. Both survival and performance, require coping with specific environmental stressors by adaptive biological processes of various kinds, which are adaptation, acclimatization, acclimation, and habituation (Folk, 1966; IUPS, 1987; Gunga, 2015). Adaptation represents an evolutionary process as a result of natural selection occurring over generations, which results in the expression of certain genes that optimize functions (genetic adaptation). It also occurs in the course of the life-span of an organism, where specialized organ functions are required (phenotypic adaptation). Acclimatization is initiated by exposure to extreme natural environments of previously not-exposed individuals and occurs gradually within days to weeks, sometimes even months, enabling maintenance of performance. In contrast to acclimatization, acclimation involves adaptive processes induced by exposures to habitats, where specific types of extreme conditions are simulated in order to achieve acclimatization for later exposure to naturally occurring extreme habitats. Finally, habituation defines the process of reducing physiological and psychological stress responses upon repeated stimuli, i.e., improved tolerance. Adjustments include physiological and morphological, as well as behavioral responses, of which in the following some short examples will be given.

*Physiological responses*, for instance help maintaining homeothermy upon thermal stress (Moen, 1968). In heat stress, cooling mechanisms include increased skin blood flow and sweating and at the same time circulatory measures are initiated to maintain central blood pressure, e.g., by an increase in plasma volume and cardiac output (Taylor, 2014). In cold stress, heat loss is prevented by peripheral vasoconstriction and heat production by shivering and uncoupled mitochondrial activity (Manou-Stathopoulou et al., 2015). In hypoxic stress, i.e., high altitude, hyperventilation, hemoconcentration, and stimulated erythropoiesis are physiological responses to warrant oxygen delivery to tissues (West, 2006, 2017).

*Morphological adjustments* in extreme thermal stress for instance include alterations in the body mass to body surface ratio and in the amount of subcutaneous fat mass. Both are increased in cold environments (Newman, 1961), which makes sense due to the heat-conserving properties. Larger size of chest and lung is found in high altitude natives (Newman, 1961), eventually enabling higher ventilatory capacity.

*Behavioral adjustments* in turn play an important role for life of modern humans in extreme environmental conditions. They improved with advances in technology and include appropriate clothing, shelter, air conditioning, and oxygen enrichment of facilities (Makinen, 2010; West, 2015, 2017).

In contrast to acute responses, long-term adjustments require altered cellular functions, which are the basis of optimized performance of organs and the entire organism, and which include altered regulation of metabolic pathways as well as altered gene and protein expression. Typical examples are the heat response induced by heat shock proteins (HSP) (Michel and Starka, 1986), uncoupling proteins (UCPs) in brown adipose tissue for heat production (Cannon and Nedergaard, 2004), and the adjustments of aerobic and anaerobic metabolism to hypoxia, which is governed by a family of hypoxia-induced transcription factors (HIFs) (Semenza, 2012). It needs to be pointed out that none of these is truly specific to the respective environmental stressor, because all of them also respond to other stressors such as oxidants, inflammation, and cancer.

Although a complete overview on adaptations at various levels by far exceeds the scope of this review at this point, the examples of adaptive responses discussed above may highlight their wide variety and complexity, as well as the capacity of physiological, morphological, and behavioral means of humans to adapt, which is best seen by studying natives living in remote, extreme habitats. Better understanding of these adaptive processes will provide the knowledge on survival, performance and of disease states of humans living in extreme terrestrial environments and how to survive and to achieve optimal performance if not being a native. And it is of particular importance when the organism has to cope not only with a single but with combinations of stressors (Gibson et al., 2017).

## Humans Exposed to Extreme Environmental Temperatures

Humans are homothermic, and to ensure optimal physiological function, body temperature has to be regulated within a relatively narrow range, i.e., 35–37.5°C. When exposed to extreme environmental temperatures the thermoregulatory system is challenged to maintain a stable core temperature such as by preventing heat loss and increased thermogenesis in the cold, and by removing heat when the core temperature is increased. Specific morphological, physiological and behavioral adjustments enable people to live a normal life in such extreme areas.

### Adaptive Responses to Cold

#### Adaptation of Natives to Cold Environments

Early studies on human cold adaptation demonstrated lower ratios of body surface area to body mass of people living in colder regions protecting them from extensive heat loss (Ruff, 1994). This relationship, however, has become less significant with changing nutrition pattern over time (Newman, 1961; Katzmarzyk and Leonard, 1998; Makinen, 2010). People living in cold environments exhibit different types of cold adaptation depending on the climate and lifestyle. Types of adaptation can be distinguished by physiological responses to cold exposure, namely, hypothermic, insulative, metabolic, or mixed (Scholander et al., 1958a; Makinen, 2010) (**Table 1**). A hypothermic response is characterized by a more pronounced drop in core temperature when compared to non-acclimatized individuals. A decrease of the skin temperature indicates an insulative response, and metabolic thermogenesis (shivering and non-shivering) a metabolic response (Makinen, 2010). Insulation may have a passive (subcutaneous fat) and an active (vasoconstriction of the skin and peripheral tissues) component.

**Table 1:** Most important adaptive responses to acute, repeated and chronic exposure to cold and heat.

COLD			HEAT	
		**Acute exposure**		
Cutaneous vasoconstriction			Cutaneous vasodilation	
Shivering			Sweating	
		**Few short-term exposures**		
Habituation:	reduced cold shock response		Plasma volume expansion	
			Enhanced sweating and cutaneous blood flow	
		**Long-term exposures, natives**		
Various forms of adaptation (individual or mixed)				
Insulative:	Vasoconstriction of skin and peripheral tissue		Evaporative:	Optimal evaporation response with minimized sweat dripping
	Subcutaneous fat increase			Skin blood flow adjustments
Metabolic:	Shivering and non-shivering thermogenesis		Metabolic:	Metabolic rate adjustments (?)
Hypothermic:	Drop in core temperature		Hyperthermic:	Higher resting core temperature (?)

For example, Australian Aborigines live in a semi-desert, and they sleep barely covered and without shelter at an ambient temperature of about 4°C at night. They do not increase metabolic heat production during exposure to the cold and decrease body temperature compared to non-acclimatized individuals indicating a hypothermic-insulative adaptation (Scholander et al., 1958b). Kalahari Bushmen demonstrate somewhat different responses without shivering when sleeping at about 0°C. They usually sleep around fires generating a fairly comfortable ambient temperature. They show a small increase in metabolic heat production but a less pronounced decrease in body temperature indicative of insulative adaptation (Wyndham and Morrison, 1958). Whereas rather mild cold and low caloric intake results in insulative adaptation (Australian Aborigines), metabolic adaptation is typically associated with exposure to more severe cold and high caloric intake (Arctic Inuit) (Leonard et al., 2002; Makinen, 2010). Metabolic adaptation was also seen in Alacuf Indians of Tierra del Fuego who demonstrate a much higher metabolic heat production during cold exposure when compared to non-acclimatized individuals (Hammel, 1960). Beside behavioral responses, extreme cold environment triggers also shivering and rather mild cold conditions provoke non-shivering thermogenesis (Janský, 1973). Cold-induced recruitment of brown fat represents an important source for non-shivering heat production (van der Lans et al., 2013). An up to 19% higher basal metabolic rate has been shown for indigenous populations living in polar regions compared to people living in temperate climates (Leonard et al., 2002). The higher basal metabolic rate seems to be linked to seasonal thyroid responses in these populations. In summary, people native to cold areas exhibit primarily metabolic or insulative types of adaptation depending on the severity of cold exposure and energy intake. However, behavioral responses, including life in houses, heating, and clothes, provide much more protection than physiological responses alone.

#### Adaptation Due to Repeated Cold Exposure

Various occupations, sports and adventures are associated with seasonal and repeated cold exposures over long time periods (months to years). Examples are pearl divers in Korea, winter swimmers, participants in polar and high-altitude expeditions, skiing instructors, mountain guides, and downhill and nordic ski racers. It is difficult to distinguish whether adaptive effects arose from stimuli like cold, altitude, or high-intensity exercise. Winter swimmers (Vybíral et al., 2000) and female pearl divers from Korea (Tipton and Bradford, 2014), who are primarily exposed to cold water, may exhibit two or all three forms of cold adaptation (insulative, hypothermic, and metabolic) and, additionally, local cold adaptation. In winter swimmers shivering occurred considerably later during cooling when compared to not cold-adapted subjects, which was explained by reduced heat loss, more pronounced non-shivering thermogenesis, but less total heat production (Vybíral et al., 2000). Insulative adaptation was demonstrated in the cold-adapted during swimming, and hypothermic adaptation occurred when only sitting in cold water (Tipton and Bradford, 2014). Improved cold tolerance has also been shown in pearl divers daily immersed in cold water for several hours (Hong, 1973). Besides insulative, hypothermic and metabolic forms of adaptation those women also exhibited reduced heat flux in the limbs indicating local cold adaptation. Interestingly, however, pearl divers lose their cold adaptation within 3–5 years when wearing wet suits (Park et al., 1983).

#### Acclimation and Habituation to Cold Environments

In contrast to the excellent capabilities of humans for heat acclimatization, potential acclimatization to cold remains a matter of discussion (Brazaitis et al., 2014a). Acclimation studies are most appropriate to investigate type and time course of cold responses under standardized conditions and in individuals/populations of interest. Most studies have been performed by cold water immersion due to the high conductivity and thermal capacity of water compared to air. Cold water immersion elicits typically a cold shock response characterized by sympathetic activation, hyperventilation and tachycardia (Castellani and Tipton, 2015). However, repeated immersions in cold water (habituation) considerably decreases these responses, and this depression is lasting for 7–14 months (Tipton et al., 2000) (**Table 1**). Biological mechanisms involved in habituation of the cold shock response are still unclear. Mental processes seem just as involved as cold stimuli *per se* and the frontal cortex area seems to play a pivotal role (Glaser and Griffin, 1962; Castellani and Tipton, 2015). Even a small number of repeated short cold exposures (e.g., 3 min × 60 min) resulted in less discomfort, delayed, and reduced intensity of shivering associated with lower skin temperature, which is indicative for insulative adjustment (Makinen, 2010). Longer durations (90 min – 3 h) of cold water immersion (10–18°C) resulted in insulative or combined metabolic insulative acclimation (Young et al., 1986). It has been suggested that skin cooling is sufficient to provoke the vasoconstrictor response, whereas a decrease (0.8°C) in core temperature seems to be necessary to induce sympathetic activation (Makinen, 2010).

Types of acclimatization/acclimation to cold air depend mainly on the duration of exposure. Whereas exposures for up to about 1 h cause habituation with a later onset of shivering and maintenance of skin and core temperatures (Hesslink et al., 1992; Makinen, 2010), longer exposures (3 h to days) result in hypothermic habituation (Mathew et al., 1981). A few studies suggest that chronic cold exposure (weeks), e.g., during camping in remote areas without sufficient cold protection, triggers metabolic acclimatization (Scholander et al., 1958a).

Studies have typically been performed under resting conditions. It is remarkable that exercise during the cold exposure seems to blunt some of the acclimation effects, e.g., the hypothermic acclimation observed during rest (Launay et al., 2002). Another study demonstrated increased susceptibility to hypothermia after multiple days of severe exertion in cold environment (Castellani et al., 2001). This topic has not been investigated extensively and urgently needs further attention.

#### Physiological and Pathophysiological Responses to Acute Cold Exposure

Acute cold exposure causes powerful autonomic homoeostatic responses in order to prevent heat loss and to maintain core body temperature. Cutaneous vasoconstriction and thermogenesis (**Table 1**), primarily by skeletal muscle contractions, are most important and are particularly powerful when cooling involves both superficial and deep thermoreceptors (Stocks et al., 2004). Thermogenesis may be achieved by voluntary behavioral responses, e.g., physical activity, but occurs involuntarily by shivering. The magnitude of the effector responses to cold are varying depending on the severity and duration of exposure, physical activity and clothing, and individual characteristics such as age, gender, and body composition (Stocks et al., 2004). When these responses are not sufficient to maintain core temperature, the risk of hypothermia will occur if not protected by appropriate clothing (Burtscher et al., 2012). Moreover, peripheral vasoconstriction may also enhance the risk of cold injury and impair manual dexterity. The occurrence of freezing and non-freezing cold injuries depends on the severity and duration of cold exposure (Vale et al., 2017) and may predispose for future cold injury (Gorjanc et al., 2018). Systemic blood pressure (systolic and diastolic) typically increases, and heart rate may increase, e.g., when only legs are cold exposed, or decrease with whole body exposure, again depending on the severity of cold (Korhonen, 2006). The augmentation of the double product (systolic blood pressure × heart rate) indicate elevated myocardial oxygen consumption. This fact may in part explain the cold-induced provocation of angina attacks in patients suffering from coronary artery disease, especially in those with an abnormal baroreceptor function (Marchant et al., 1994). In addition, elevated blood pressure and centralization of blood might cause an increase of diuresis (cold-induced pressure diuresis). The primary effect of cold air on the respiratory system is to decrease minute ventilation and chemosensitivity which is not really efficient in humans. More importantly, however, bronchoconstriction, airway congestion, secretion and reduced mucociliary clearance may compromise pulmonary mechanics in subjects with cold- or exercise induced asthma (Giesbrecht, 1995). In addition, acute cold stress was shown to impair vigilance, overall mood, motor, and cognitive performance (Brazaitis et al., 2014b). However, most of these responses to acute cold exposure diminish or even disappear with habituation or acclimation.

#### Cellular Responses to Cold Exposure

Prevention of cooling can be achieved by increasing heat production by excessive breakdown of stored nutrients such as fat, which is called non-shivering thermogenesis. Prolonged cold exposure results in an increase in PGC1α protein expression, which in turn stimulates mitochondrial biogenesis, e.g., in skeletal muscle (Chung et al., 2017). In white and in brown adipose tissue the cold-sensing receptor TRPM8 seems to initiate increased mitochondrial thermogenesis (Frontini and Cinti, 2010; Rossato et al., 2014) by up-regulating mitochondrial UCPs (e.g., UCP1). In brown adipose tissue UCP1 uncouples mitochondrial oxygen consumption from oxidative phosphorylation resulting in heat production, which is an important means to regulate body temperature in cold ambient temperatures, in particular in small mammals and human infants (Cannon and Nedergaard, 2004). Such mechanism also occurs in beige adipose tissue (Shabalina et al., 2013), whose formation is stimulated in the cold (Fisher et al., 2012) or by caloric restriction (Fabbiano et al., 2016). Notably, acute exposures (3 h) of healthy humans to cold temperatures (7°C) seems to not elicit changes in skeletal muscle gene expression (Zak et al., 2017). Therefore, it has been discussed that exercise might be a necessary accompanying condition to induce expression of respective genes when acutely exposed to extreme environmental temperatures.

#### Clinical and Practical Relevance of Cold Acclimation

The question arises which lessons can be learned from the present findings on cold adaptive responses and how does knowledge impact on general health and/or on performance in profession and sports practiced in cold environments. Studies addressing these questions are rare and thus the following suggestions are sometimes speculative.

It seems reasonable that cold habituation accompanied by more comfortable feeling and delayed shivering responses would be beneficial during work and exercise in cold conditions. However, the assumption that reduced thermal responses of hand/fingers would enhance dexterity and thus the ability to work/exercise under cold ambient conditions has not been confirmed (Muller et al., 2014). Moreover, repeated intense exercise of outdoor winter athletes seems to provoke exercise-induced asthma obviously without beneficial acclimation effects (Carlsen, 2012). Furthermore, acute cold exposure such as exposures to other extreme environmental conditions, are associated with increased sympathetic activity and the risk of cardiac events in susceptible subjects (Cordioli et al., 2000). However, cold acclimation has been shown to blunt sympathetic stimulation likely representing an effective prophylactic measure (Mäkinen et al., 2008). A recent study reported promising effects of cold acclimation on other potentially health-related effects (van der Lans et al., 2013). Individuals subjected to intermittent cold exposures for 10 days (15–16°C; 2–6 h per day) felt more comfortable and reported less shivering in the cold. The authors demonstrated pronounced recruitment of brown adipose tissue and increase in energy expenditure through non-shivering thermogenesis which might be used to counteract the current obesity epidemic (van der Lans et al., 2013).

### Adaptive Responses to Heat

#### Adaptation of Natives to Hot Environments

Living for generations in hot environments (i.e., indigenous dwellers) leads to evolutional heat adaptation aimed at reducing physiological strain in the hot environment (Taylor, 2014). These adaptations include morphological and genetic changes as well as functional adjustments. As such, indigenous inhabitants of hot regions show some apparent differences to dwellers of temperate environments that might reflect adaptations stimulated by the unique environment these residents live in (Lambert et al., 2008; Taylor, 2014). In this regard morphological adaptations have been reported with people from the hottest regions (i.e., South Asia, Africa, India, and Australia) showing the largest surface area to body mass ratio (Taylor, 2006; Tipton et al., 2008). Of course, factors other than heat exposure, as for example diet might have determined characteristic body composition evolution as well. Nonetheless, the relationship between surface area and body mass may be seen as an important determinant of heat balance (Lambert et al., 2008; Taylor, 2014). This morphological shape favors dry heat exchange known to be the most efficient way of heat dissipation (Tipton et al., 2008) and may reduce reliance upon evaporation in hot and humid conditions (Taylor, 2014). However, in conditions where air temperature approaches and exceeds skin temperature, dry heat loss is minimized or even reversed leaving evaporative cooling the main means of heat dissipation (Tipton et al., 2008). Here, the larger body surface area of indigenous people augments the area available for evaporation, thus improving thermoregulation in hot dry environments (Fudge et al., 2008). With respect to evaporation, another morphological difference between indigenes of hot climates and people from a temperate area could be a variation in the distribution and number of eccrine sweat glands (Taylor, 2006, 2014). Indeed, some early studies reported greater glandular density in indigenous groups, yet according to Taylor (2006, 2014) racial differences do unlikely exist as the majority of studies did not find difference in the number of sweat glands (Taylor, 2006, 2014). Besides number, the ability to activate the sweat glands determines sudomotor capacity (Taylor, 2006). An increased sweating rate is one of the alterations occurring with short term heat acclimation as outlined below (Périard et al., 2016). Thus, it might be anticipated that if effective, these short-term adjustments could also lead to long-term adaptations. However, since evaporative cooling is energetically wasteful, particularly in hot and humid conditions, such adaptations might even be detrimental leading to excessive water loss, which may challenge fluid homeostasis (Taylor, 2006, 2014; Tipton et al., 2008; Lee et al., 2009). Accordingly, in indigenous people of hot and tropical countries lower sweat response relative to people of cooler countries have been suggested (Wijayanto et al., 2011; Taylor, 2014; Lee et al., 2016), indicating that these dwellers possible adopt an optimal evaporation response with minimized sweat dripping (Tipton et al., 2008) (**Table 1**). Additionally, a higher resting core temperature in the warm has been reported in some studies, which from an ecological viewpoint might be helpful to reduce sweat loss and water consumption (Nguyen and Tokura, 2002; Saat et al., 2005; Wakabayashi et al., 2011; Wijayanto et al., 2012) and additionally may reduce the total amount of heat that must be dissipated (Lambert et al., 2008). Yet, in contrast, also lower core temperatures at rest and during sleep have been described in indigenes, which have been linked to a supposed lower resting metabolic rate as outlined below (Lee et al., 2009; Taylor, 2014). Furthermore, studies reported lower skin blood flow of indigenes compared to un-acclimatized Caucasians, which allows skin temperature to rise with the effect of a reduced exogenous heat gain while simultaneously enhancing evaporation (Tipton et al., 2008). These adjustments may contribute to the smaller increase in core temperature during heat exposure found in heat-adapted people (Wakabayashi et al., 2011, 2014).

Another possibility to deal with the hot climate would be to reduce resting metabolic rate and thus body’s heat production (Tipton et al., 2008; Taylor, 2014). This would be of particular advantage in conditions where dry heat loss is challenged and evaporative cooling is critical due to the danger of dehydration (Taylor, 2014). However, even though an inverse relationship between basal metabolism and monthly mean ambient temperature (Hori, 1995) as well as a reduced basal metabolic rate after extended heat exposure were described, data seem more likely to indicate that such differences between ethnic groups do rarely exist (Tipton et al., 2008; Taylor, 2014). Here, it is necessary to consider that in free-living conditions distinguishing between true physiological adaptations and behavioral modifications (i.e., exercise avoidance) may be challenging (Taylor, 2014). The former description generally outlines the adaptations to heat exposure ignoring that daily living includes physical activity and exercise as well. During exercise, metabolic heat production can be increased several-fold which challenges the body’s ability to dissipate heat even more. Efficient movement and exercise patterns thus could be of considerable benefit when exercising in a hot environment (Tipton et al., 2008; Taylor, 2014). Accordingly, a greater metabolic efficiency and a shift of substrate use favoring lipid oxidation over carbohydrates after heat adaptation have been reported (Kirwan et al., 1987; Weston et al., 2000; Taylor, 2006, 2014; Tipton et al., 2008). However, when considering movement efficiency also mechanical and anthropometric next to the metabolic properties need to be addressed (Marino et al., 2004). Moreover, in people of a tropical environment specific distribution of blood to the acral area during exercise was reported, which is suitable for heat loss as the extremities have a large surface area to mass ratio and its containing blood may mix with warm core blood (Magalhães et al., 2010; Lee et al., 2011; Wakabayashi et al., 2011). Additionally, smaller fluid losses and reductions in plasma volume during exercise were reported in some (Saat et al., 2005; Wakabayashi et al., 2014) but not all investigations (Lee et al., 2011; Wakabayashi et al., 2011) dealing with people living in the tropical environment. An increased body fluid conservation would be of advantageous by aiding to effective heat dissipation (Saat et al., 2005; Wakabayashi et al., 2014).

#### Acclimatization/Acclimation and Habituation to Hot Environments

In contrast to the strategies adopted by indigenes, people not adapted to heat and acutely exposed to such conditions may experience different adjustments yet enabling them to cope with the hot environment. Heat acclimatization/acclimation has a relatively fast time course with increased heat tolerance and improved exercise performance in a hot environment occurring after only 5–14 days of heat exposure (Guy et al., 2014, 2016; Karlsen et al., 2015; Périard et al., 2015; Racinais et al., 2015; Neal et al., 2016). The timeline can be categorized as short-term (<7 days), medium term (8–14 days), and long-term acclimation (>14 days) (Périard et al., 2015). The mechanisms responsible for the favorable effects include plasma volume expansion and lowering of submaximal heart rate, occurring within 3–6 days (Neal et al., 2016; Périard et al., 2016). Additionally, enhanced sweating and cutaneous blood flow responses (completed after 10–14 days) together with developing thermal tolerance through heat shock response contribute to improved cardiovascular stability and exercise performance (Horowitz, 2016; Périard et al., 2016) (**Table 1**).

#### Physiological and Pathophysiological Responses to Acute Heat Exposure

Passive heat stress may induce hyperthermia and cause pronounced strain on the cardiovascular system. Large increases in sympathetic neural activity, heart rate and left ventricular contractility, coupled with reductions in central blood volume, left ventricular filling pressures and cerebral perfusion have been reported (Crandall and Gonzalez-Alonso, 2010; Akerman et al., 2016). Additionally, increased glycogenolysis and oxidative stress levels as well as different neuroendocrine responses may occur (Akerman et al., 2016). Physical exercise in hot conditions with or without dehydration may also induce hyperthermia. This causes cardiovascular strain which limits exercise performance and may even lead to serious heat associated health conditions (Crandall and Gonzalez-Alonso, 2010; Yankelson et al., 2014; Brocherie et al., 2015; Racinais et al., 2017), which include heat rash, syncope, heat cramps, heat exhaustion, and heat stroke (Howe and Boden, 2007; Brocherie et al., 2015; Racinais et al., 2017). The responses of subjects exposed to a hot environment will depend on type, duration and intensity of exercise, as well as additional factors (i.e., related to body composition, clothing or other weather independent variables) (Brocherie et al., 2014). With respect to exercise performance, a reduction in cardiac output, stroke volume, arterial pressure and blood flow to the brain, skin and exercising muscles have been observed prior to exhaustion (Crandall and Gonzalez-Alonso, 2010; Akerman et al., 2016). Hyperthermia may also impair central as well as peripheral neuromuscular function (Racinais et al., 2017).

#### Cellular Responses to Heat Exposure

A common response of cells to acute heat exposure is the increased expression of HSP (heat shock response). However, the heat shock response is not only triggered by temperature increase but also by other stresses like oxidative stress or toxic substances like ethanol (Michel and Starka, 1986). HSPs are molecular chaperones which prevent cellular structures from damage. The critical regulator for the heat shock response is the transcription factor heat-shock-factor (Hsf1), which binds to the heat shock element (HSE) on DNA thereby initiating the transcription of HSPs (Wu et al., 1986). The protective effect of HSPs lies mainly in the prevention of high-temperature-induced misfolding of proteins and removal of damaged proteins to prevent toxic effects (Parsell and Lindquist, 1993; Henstridge et al., 2016; Zhu et al., 2016).

Another cellular response to increased temperature is an adjustment of cellular energy metabolism. However, mechanisms are not well established in humans. In insects, however, a shift toward anaerobic glycolysis during exposure to heat has been described (Malmendal et al., 2006). In ectoderm vertebrates a down-regulation of cytochrome c oxidase and citrate synthase has been demonstrated (Seebacher, 2009), which was accompanied by reduced activity of PGC1α, which is a bio-energetic master-regulator and controls mitochondrial metabolism (Scarpulla, 2011; Vincent et al., 2015). This is in line with observations on reduced expression of mRNAs related to mitochondrial proteins in humans exercising in hot environments as opposed to room temperature (Heesch et al., 2016). However, it appears that heat as a sole stimulus is ineffective, but that exercising in the heat is required to induce the respective metabolic adjustments in skeletal muscle (Zak et al., 2017).

#### Clinical and Practical Relevance of Heat Acclimatization/Acclimation

As mentioned before the adjustments found after heat acclimatization/acclimation may differ to some extent to the adaptations found in indigenous dwellers of hot environments. In contrast to the body water saving strategies adopted by indigenes, short term heat acclimation increases sweating rate (Périard et al., 2016) in order to enhance evaporative cooling which, however, may also lead to hypohydration (Akerman et al., 2016). Hypohydration and heat strain can reduce central venous pressure and stroke volume, increase oxidative stress and may induce several neuro-endocrine responses (e.g., increased plasma levels of renin activity, aldosterone and catecholamine) (Francesconi et al., 1983; Wright et al., 2010; Akerman et al., 2016). In addition, hypohydration (>2–3% of body mass) may augment effort perception and negatively affect exercise performance, especially in hot environments (Sawka et al., 2015; Nuccio et al., 2017). So far it is not clear if some degree of dehydration is necessary for optimal heat acclimation (Akerman et al., 2016), yet a recent study found that the time course and magnitude of the acquisition of heat acclimation are largely unaffected by permissive dehydration or maintaining euhydration (Neal et al., 2016). Nonetheless, it seems advisable to reduce the negative consequences of severe hypohydration in hot environments and during heat acclimation. With respect to physical performance in hot environments, pre heat acclimation allows individuals to complete tasks in the heat that prior to acclimation were difficult or even impossible (Périard et al., 2015). Thus, if unaccustomed people have to perform or work in hot environments a prior short-term heat acclimatization/acclimation program lasting for at least one but ideally 2 weeks and including exercise sessions (e.g., every second day for 60–90 min) is advisable to minimize the heat dependent performance loss (Brocherie et al., 2015; Périard et al., 2015).

## Life in a Hypoxic Environment

Impaired oxygen supply compromises cellular functions and thus performance. It can be caused by environmental hypoxia such as at high altitudes (HA), where the partial oxygen pressure (PO_2_) is decreased in proportion to the lower ambient pressure. In patients, common reasons causing hypoxia are hypoventilation, impaired oxygen diffusion in the lungs (e.g., pulmonary edema), anemia, and impaired cardio-vascular function. Here we will focus on major mechanisms of adjustment of tissue oxygen supply to hypoxia at HA, which are ventilation, alveolar diffusion and oxygen transport in blood, and we will dissect differences between HA natives showing genetic and phenotypic adaptation, and lowlanders during sojourns at HA, which represents acclimatization. Differences between those mechanisms are demonstrated best by the fact that deterioration in physical performance with increasing altitude is much less pronounced in HA natives than in naïves within the 1st days and even weeks of their sojourns (Brutsaert, 2008). Also, aspects of acclimation will be discussed, which are of significance for performance at HA and for the prevention of acute mountain illnesses that are often observed in not-acclimatized lowlanders (Bärtsch and Swenson, 2014).

### Adaptation of Natives to High Altitude

The HA natives show improved hypoxia-tolerance in comparison to sojourners, which is characterized by a variety of physiologic and morphologic adjustments. However, there are differences among HA natives of different ethnicity.

#### Ventilation and Alveolar Gas Exchange

All HA natives hyperventilate relative to lowlanders at low altitude (**Table 2**). However, the degree of hyperventilation to a defined hypoxic stimulus (hypoxic ventilatory response, HVR) varies considerably among different ethnic groups. Tibetans seem to have an elevated HVR (Curran et al., 1995), whereas it seems blunted in Andeans (Beall et al., 1997) and in Sherpas (Santolaya et al., 1989) implying differences in the sensitivity of the peripheral chemoreceptor (Moore, 2000).

**Table 2:** Summary of adaptive responses to hypoxia.

Hypoxia
**Acute exposure (a few hours)**
Hyperventilation Pulmonary arterial vasoconstriction
Peripheral vasodilation Increased cardiac output
**Exposure for days to months (intermittent/permanent)**
Hyperventilation Pulmonary arterial vasoconstriction
Low-to-normal cardiac output Decreased plasma volume: increased hematocrit, hemoglobin Stimulated erythropoiesis: increased total hemoglobin mass
**High altitude natives**
Hyperventilation Increased lung volume and diffusion capacity Pulmonary arterial vasoconstriction
Low-to-normal cardiac output Decreased plasma volume: increased hematocrit, hemoglobin Stimulated erythropoiesis: increased total hemoglobin mass

Because in HA natives hyperventilation begins with their first breath and is maintained throughout their life, it is likely the main mechanism causing extended growth of the lungs resulting in greater forced vital capacity and increased lung volumes and diffusion capacity in HA natives compared with Han Chinese (East Asian ethnic group) living in the Tibet and with sojourners to the Andes, who moved to HA as adults and lived there for several years (Jones et al., 1992; Brutsaert et al., 2004). Similarly, larger lung volumes and increased diffusion capacity has been found in rodents and dogs raised in hypoxia. Animals raised in normoxia that moved to hypoxia for several years did not acquire larger lungs (Brutsaert, 2016). It has to be noted, however, that also lifestyle favors the development of larger lungs in HA natives, because many study subjects were physically much more active than their low-land-counterparts (Kashiwazaki et al., 1995).

Hypoxia-induced vasoconstriction of small pulmonary arteries (Euler-Liljestrand reflex) seems to be blunted in Tibetans (Groves et al., 1993), which has been discussed to protect from augmented fluid filtration into the alveoli and thus from interstitial and alveolar edema, and to favor alveolar oxygen diffusion in HA natives relative to lowlanders with high pulmonary capillary pressure at HA. In fact, a decreased alveolar-to-arterial oxygen difference has been found in HA natives (Lundby et al., 2004), but results are conflicting.

Together, these results indicate phenotypic adaptation with improved alveolar oxygen diffusion and higher arterial oxygen saturation (SaO_2_) at rest and during exercise (Wagner et al., 2002; Lundby et al., 2004; Brutsaert, 2016).

#### Oxygen Transport by Hemoglobin

An increased oxygen affinity of hemoglobin (Hb-O_2_-affinity) in hypoxia is favorable, because it results in higher SaO_2_ at a low PO_2_ than low oxygen affinity (Mairbäurl and Weber, 2012). It is unclear, however, whether adjustments at this level occur in HA natives. No differences in Hb-O_2_-affinity have been found between sojourners and Andeans (Lundby et al., 2006), and between sojourners and Sherpas (Samaja et al., 1979). However, increased Hb-O_2_-affinity in HA natives has also been reported (Balaban et al., 2013; Simonson et al., 2014). This is in line with increased Hb-O_2_-affinity in animals native to HA relative to their lowland counterparts (Petschow et al., 1977).

#### Amount of Hemoglobin

An increased hemoglobin concentration ([Hb]) is of advantage in hypoxia because it increases the oxygen content of arterial blood to compensate for decreased SaO_2_. Most HA natives show an increased [Hb] in blood (**Table 2**). The degree of elevation directly relates to the absolute altitude. However, [Hb] varies considerably among different ethnical groups living at comparable altitude. Most data were obtained in the Andes and on Han-Chinese living on the Tibetan plateau, where a pronounced increase in [Hb] has been found (e.g., Hurtado et al., 1945; Cosio and Yataco, 1968; Winslow and Monge, 1987; Wu et al., 2014). However, similar to the animal kingdom, where yaks and alpacas have lower [Hb] than their lowland-relatives, cows and llamas, respectively, when exposed to HA (Bouverot, 1985), also some human populations living at HA have only mildly elevated [Hb]. Those are Tibetans (Simonson et al., 2010) and Ethiopians (Beall et al., 2002), whose [Hb] is a few g/dl lower than that of Andeans and Han Chinese (Beall et al., 1998), and their [Hb] actually is close to normal sea-level-values despite living at altitudes >3500 m. Differences exist even among Andean populations, where Quechuas have lower [Hb] than Aymaras (Arnaud et al., 1981). In any of the aforementioned populations the altitude-related increase in [Hb] exist both in men and in women (Wu et al., 2005; Gassmann et al., unpublished data).

Though most studies have been performed on adult individuals, a few studies indicate that [Hb] is already elevated during childhood in Han-Chinese (Wu et al., 2005) and in Quechuas (Garruto et al., 2003) living at altitudes between 4200 and 4500 m, whereas [Hb] of Tibetan children living at HA is only slightly higher (Wu et al., 2005) than that of lowland-children (Fulwood et al., 1982) of ages between 6 months and 15 years, both in girls and in boys. Data on newborns are sparse (Moore et al., 1982; Niermeyer et al., 1995), but [Hb] appears to be within the range of lowland-newborns, with a tendency of lower [Hb] in Tibetan than in Han Chinese newborns in Lhasa (Niermeyer et al., 1995).

The mechanisms explaining the different patterns in [Hb] in HA natives of different ethnicity are not fully understood. The lower [Hb] in Tibetans than in Han Chinese is associated with putatively advantageous polymorphisms of EPAS1 (HIF-2α) (Beall et al., 2010), the prolyl hydroxylase EGLN1 and PPARα (Simonson et al., 2010) in Tibetans, as well as of the thyroid hormone receptor THRB and, marginally, also of EPAS1 in Ethiopians (Scheinfeldt et al., 2012), which are all associated with low [Hb] thus indicating genetic adaptation.

There is a clear increase in total hemoglobin mass (tHb) with decreasing SaO_2_ in North Americans residing at different altitudes (Weil et al., 1968). However, a significant increase in tHb was only found when PaO_2_ dropped below ∼70 mmHg. This is consistent with data showing that Bolivians living at 2600 m did not have an elevated tHb (Schmidt et al., 2002; Böning et al., 2004) indicating that the stimulus of chronic hypoxia at moderate altitudes was not strong enough to induce polycythemia. In contrast, tHb was elevated by ∼15% in La Paz (3600 m) (Wachsmuth et al., 2013), and by up to 80% in residents of Cerro de Pasco (4330 m) (Sanchez et al., 1970). However, excessive high [Hb] is considered maladaptive and represents a key factor of chronic mountain sickness (CMS) first described by Carlos Monge in the Peruvian Andes (Vargas and Spielvogel, 2006).

#### Cardiac Function

Cardiac function is well maintained in HA natives; however, most reports indicate a slightly lower cardiac output, e.g., in Andeans, than sea level residents (Banchero, 1987). Interestingly, when HA natives return to sea level, cardiac output increases significantly within approximately 10 days, likely because improved venous return (Hartley, 1971).

### Lowlanders Ascending to High Altitude

Lowlanders ascending to HA without any pre-acclimatization usually do not tolerate altitudes >4000 m very well in the sense that their performance is considerably decreased in comparison to performance at low altitude and relative to HA natives (Klausen et al., 1970). Thus immediate means of acclimatization such as hyperventilation, hemoconcentration and erythropoiesis are initiated to increase total oxygen carrying capacity. Yet, low altitude performance will not be fully regained while at HA.

#### Alveolar Ventilation and Oxygen Diffusion

Decreased oxygen content in arterial blood is detected by carotid bodies, which generate signals stimulating alveolar ventilation. Hyperventilation at HA slightly raises the alveolar partial pressure of oxygen (PAO_2_) at the expense of decreased arterial partial pressure of carbon dioxide (PCO_2_) and respiratory alkalosis, and slightly elevates SaO_2_ as long as alveolar oxygen diffusion is not impaired. However, the degree of hyperventilation to a defined hypoxic stimulus varies considerably among individuals, where those with the lowest HVR are the least hypoxia-tolerant ones indicated by increased susceptibility to HA pulmonary edema (HAPE) (Hohenhaus et al., 1995). Sojourners to HA also show pronounced hypoxic pulmonary vasoconstriction (Sylvester et al., 2012) (**Table 2**), which favors alveolar filtration and subclinical edema, and which, if exaggerated vasoconstriction occurs, might result in HAPE with massively impaired *trans*-alveolar oxygen diffusion (Swenson et al., 2002). Even after long-term sojourns lowlanders did not acquire the improved gas exchange observed in HA-natives (Dempsey et al., 1971).

#### Cardiovascular Responses

Due to the decreased arterial oxygen content cardiac output increases in order to maintain oxygen delivery to the periphery. At rest, the increase is mainly due to an increased heart rate, while the stroke volume is decreased, likely due to a decreased venous return because of peripheral vasodilation (Alexander and Grover, 1983). However, with prolonged stay at HA, cardiac output decreases and might even fall below low altitude values. Although cardiac output for the same work load is higher at HA than at low altitude, stroke volume remains decreased (Alexander et al., 1967). Maximal heart rate is decreased because of down-regulation of adrenergic β-receptors (Kacimi et al., 1992). In general, it has been assumed that cardiac function is preserved in sojourners to HA (Reeves et al., 1987).

#### Oxygen Affinity of Hemoglobin

Hyperventilation decreases arterial CO_2_ and results in respiratory alkalosis. Together, both effects increase the Hb-O_2_-affinity which improves oxygen binding by hemoglobin even at low arterial PO_2_ (Mairbäurl and Weber, 2012). Furthermore, elevated levels of organic phosphates, such as 2,3-DPG, increase the Bohr effect on Hb-O_2_-binding which favors the release of oxygen from its Hb-bond in peripheral tissues (Mairbäurl and Weber, 2012).

#### Amount of Hemoglobin

It has long been known that [Hb] increases in lowlanders sojourning at HA (Viault, 1891). The nature of the increase can be dissected into two phases: A rapid decrease in plasma volume is observed upon exposure of lowlanders to HA (Sawka et al., 2000; Siebenmann et al., 2017). It results in increased [Hb] and increases the amount of oxygen transported per unit-volume of blood (e.g., per stroke-volume). Decreased plasma volume is maintained over months at HA (Reynafarje et al., 1959; Hannon et al., 1969). The loss of plasma water is likely caused by decreased aldosterone activity and increased levels of atrial natriuretic peptide resulting in an increased fractional Na-excretion and diuresis (Olsen, 1997), but other mechanisms may contribute as well (Sawka et al., 2000).

Hypoxia also stimulates erythropoiesis. It affects both [Hb] and tHb. Erythropoiesis is stimulated by elevated erythropoietin levels (EPO), a HIF-induced anti-apoptotic growth factor (Wenger and Kurtz, 2011). Changes in EPO follow interesting kinetics: first, a pronounced increase can be observed within a few hours after exposure to hypoxia, which is then followed by a decrease to considerably lower levels that are still above the low altitude plasma concentration. Both, initial peak and steady state EPO depend on the degree of hypoxia (Wenger and Kurtz, 2011). Despite this rapid increase in EPO it takes weeks to months to gain significant amounts of new erythrocytes and increased tHb. The magnitude of increase depends on absolute altitude and duration of stay (Garvican et al., 2012; Rasmussen et al., 2013). However, there is great variability in the erythropoietic response to HA (Chapman et al., 1998; Hauser et al., 2017). This aspect is of particular importance in athletes who chose training at HA to improve aerobic performance.

Only to mention, HA (hypoxia) exposure is accompanied by reduced hunger and energy intake, which should be considered by HA climbers (Matu et al., 2018).

### Acclimation and Behavioral Adjustments: Means to Improve Performance in Hypoxia

Pre-exposure to hypoxia turns on adaptive processes and thus protects from HA illnesses such as acute mountain sickness (AMS), HAPE and cerebral edema (HACE) (Bärtsch and Swenson, 2013). It also improves performance at HA (Benoit et al., 1992; Chapman et al., 2013). This is particularly of advantage, when ascent to altitudes higher than 4000 m is planned. Repeated mountain hikes and nights spent on huts above 2500 m work very well (Bärtsch et al., 1991) although systematic studies are scarce. Results on pre-acclimatization by sleeping in hypoxic tents might also be effective (Dehnert et al., 2014).

Performance at HA can greatly be improved by breathing oxygen (Klausen et al., 1970), which is often used by climbers at extreme altitudes. Air can be enriched with oxygen in workplaces located at HA (West, 2016) and in trains crossing HA ranges (West, 2008). Positive expiratory airway pressure also improves oxygenation (Schoene et al., 1985). In addition, there are pharmacologic aids preventing AMS and HAPE which also improve performance by stimulating ventilation and by lowering pulmonary artery pressure to minimize fluid filtration into the lungs, both of which improve alveolar gas exchange (Bärtsch and Swenson, 2013).

In recent years, researchers started to examine potential cross-adaptive effects between different environmental stressors, e.g., heat, cold, and hypoxia. For instance, cross tolerance has been reported for hypoxia (altitude) after prior heat acclimation-induced diminished HSP response (Gibson et al., 2017), indicating reduced cellular stress responses and better maintenance of homeostasis at HA. Future research work is necessary to explore potential clinical/practical applications and benefits related to cross adaptation.

### Cellular Responses to Hypoxia

Reduced availability of oxygen forces cells to adjust a variety of metabolic pathways aimed at maintaining adequate levels of ATP and decreasing the ATP requirement by reducing the activity of ATP-consuming processes (Hochachka, 1986).

As with the increased EPO production, most adjustments are initiated by increasing the amounts of hypoxia inducible factors (HIFs), which is achieved by preventing its degradation by decreasing the activity of HIF-prolyl hydroxylases (Semenza, 2001; Haase, 2013). HIF-1α mainly increases the expression of glycolytic enzymes, which improves anaerobic ATP production, and, at the same time, decreases mitochondrial biogenesis and activity, even initiates mitophagy (Zhang et al., 2008), all of which appear necessary, because in hypoxia mitochondria show increased production of oxygen radicals (ROS) (Solaini et al., 2010), which potentially may damage cells (Semenza, 2007). In contrast, HIF-2α increases the expression of EPO and proteins involved in iron metabolism in order to sufficiently supply erythroid progenitors with the iron required for heme synthesis and stimulated erythropoiesis (Haase, 2013; Gassmann and Muckenthaler, 2015).

ATP-demand is decreased by decreasing pathways of synthesis of substances such as proteins, steroids, and glycogen. The mediator for those responses is activation of the adenosine-monophosphate-activated kinase (AMPK), which is a master regulator of cell metabolism (Hardie et al., 2012). Activation involves HIF-dependent and HIF-independent mechanisms. As a consequence, protein synthesis, which typically requires roughly 30% of a cells energy supply, is decreased, which helps conserve ATP for metabolic pathways required for maintaining cellular integrity such as the Na/K-ATPase (Hochachka, 1986). As a consequence, specific cellular function required for the performance of certain organs might be impaired resulting in maladaptation of the entire organism.

## Conclusion

The aspects discussed above emphasize the potential of successful adaptive and acclimatization/acclimation strategies supporting survival and performance in places exposed to extreme environmental temperatures or hypoxic stressors. In the long term, specific morphological, physiological, and behavioral adjustments enable people to live an almost normal life in such extreme areas. In contrast to the strategies adopted by indigenes, people acutely exposed to extreme conditions may experience impaired performance, at least initially, and different mechanisms of adjustment will later on enable them to cope with cold, hot, and/or hypoxic environments. However, in certain circumstances also mal-adaptive processes may occur in both indigene and acutely exposed. Individually adjusted pre-exposure to specific (simulated) environmental conditions may turn on adaptive processes, which improves performance and provides some protection from maladaptation. Finally, potential cross-adaptive effects between different environmental stressors, e.g., development of cross tolerance for hypoxia (altitude) after prior heat acclimation, may be considered.

## Author Contributions

All authors listed have made a substantial, direct and intellectual contribution to the work, and approved it for publication.

## Conflict of Interest Statement

The authors declare that the research was conducted in the absence of any commercial or financial relationships that could be construed as a potential conflict of interest.
